# Calprotectin Expression in Adventitial Layer of Cattle and Sheep *Echinococcus granulosus* sensu stricto Cysts

**DOI:** 10.3390/ijms26189236

**Published:** 2025-09-22

**Authors:** María Soledad Baquedano, Caroll Stoore, Christian Hidalgo, Ismael Pereira, Rodolfo Paredes

**Affiliations:** 1 Laboratorio Medicina Veterinaria, Escuela de Medicina Veterinaria, Facultad de Ciencias de la Vida, Universidad Andres Bello, Santiago 8370035, Chile; s.baquedanoj@gmail.com (M.S.B.); cstoorep@gmail.com (C.S.); 2 Núcleo de Investigación en One Health (NIOH), Facultad de Medicina Veterinaria y Agronomía, Universidad de las Américas, Santiago Centro 8370065, Chile; chidalgo@udla.cl; 3 Escuela de Medicina Veterinaria, Facultad de Medicina y Ciencias de la Salud, Universidad Mayor, Santiago 8200010, Chile; ismaelpereirasalas@gmail.com; 4 One Health Institute, Faculty of Life Sciences, Universidad Andres Bello, Santiago 8370035, Chile

**Keywords:** calprotectin, *Echinococcus granulosus*, adventitial layer, cystic echinococcosis, cattle, sheep

## Abstract

Cystic echinococcosis (CE) is a globally distributed zoonotic disease caused by *Echinococcus granulosus* sensu lato, forming fluid-filled cysts in humans and livestock. These cysts consist of three layers: an inner germinal layer and a middle laminar layer of parasitic origin, and an outer adventitial layer derived from the host’s immune response. The adventitial layer typically contains immune cells such as T and B lymphocytes, macrophages, and other inflammatory cells. Notably, differences have been reported in the cellular composition of this layer depending on the host species. However, the variation in calprotectin expression—a protein specific to phagocytes—between cattle and sheep CE cysts has not been previously described. This study assessed calprotectin expression using immunohistochemistry with anti-calprotectin antibodies on adventitial tissue sections from cattle and sheep CE cysts. The results showed a significantly higher calprotectin expression in the adventitial layer of cattle cysts compared to sheep. This difference was not associated with the fertility or anatomical location of the cysts. These findings suggest that the host species influences the level of calprotectin expression in the adventitial layer, contributing to our understanding of host-specific immune responses in CE.

## 1. Introduction

Cystic echinococcosis (CE) is a worldwide disease caused by the metacestode stage of the parasite *Echinococcus granulosus* sensu lato (s.l., Batsch, 1786), with higher prevalence in Southern Europe, Middle Asia, Africa, and South America of both human cases and animal hosts [1,2]. Regarding *Echinococcus granulosus* s.l. species, they are organized according to both molecular biology techniques and mammalian host: *E. granulosus* sensu stricto (s.s., Batsch, 1786), sheep; *E. equinus* (Williams & Sweatman, 1963), horse; *E. ortleppi* (Lopez-Neyra & Soler Planas, 1943), cattle; *E. canadensis* (Webster & Cameron, 1961), pig, camel, and cervids; and *E. felidis* (Ortlepp, 1937), lions [3]. Other *Echinococcus* species include *E. multilocularis* (Leuckart, 1863) and *E. shiquicus* (Xiao, Qiu, Nakao, Li, Yang, Chen, Schantz, Craig & Ito, 2005), which cause alveolar echinococcosis, and *E. vogeli* and *E. oligarthra*, which cause neotropical echinococcosis [2]. Although there are many species of *Echinococcus* described in the world, the main species present in Chile is *Echinococcus granulosus* s.s., reported in cattle and sheep [4,5]; other species reported are *E. ortleppi* [6] and *E. canadensis* [7], which were found only in 3% and 5% of the samples, respectively. It is worth mentioning that *E. granulosus* s.s. is also the most prevalent *E. granulosus* s.l. species present in humans [8].

Herbivores are intermediate hosts in the life cycle of *E. granulosus* s.l., which generates fluid-filled cysts in the liver and lungs, preferably over other organs [9]. These cysts are composed of three layers: the inner germinal layer and laminated layer from parasite origin, and the outer adventitial layer from host origin; inside, most CE cysts are filled with hydatid fluid [9,10]. The germinal layer produces protoscoleces (PSCs), which continue the parasite life cycle when ingested by the canid definitive host. The presence of PSCs defines a fertile cyst that continues the life cycle, and their absence characterizes a non-fertile cyst that does not continue the life cycle. The laminated layer is synthesized by the germinal layer cells; this extracellular matrix is the defining structure of the *Echinococcus* genus [9]. The adventitial layer comprises both the host’s innate and adaptative immune cells, and, in some cases, a fibrotic reaction.

Epidemiological data of cyst fertility in the world is diverse. Chile reports show that 79% of CE cysts are non-fertile in cattle [6]; these values are comparable to results obtained Ethiopia [11], India [12], and Brazil [13]. Conversely, in Sudan, cattle infected with *E. granulosus* s.l. demonstrated a higher (77%) fertility rate [14]. Sheep CE cysts usually are fertile. Reports in Chile show that 90% of CE cysts are fertile [15], a value which is in agreement with data from India [16], Morocco [17], Saudi Arabia [18], and Kenya [19]. Although CE cyst fertility has been recorded in epidemiological studies for decades, the molecular mechanisms responsible for non-fertile CE cysts remain unknown [20]. Since fertile and non-fertile CE cysts of the same haplotype can be found in both cattle and sheep, even within the same animal and organ [4], variables such as the intermediate host or the *E. granulosus* s.l. species cannot be considered the sole causes of non-fertile CE cysts. Nonetheless, non-fertile CE cysts have thinner laminated layers than fertile CE cysts [10], have higher amounts of nitric oxide in the hydatid fluid [21], and have a higher apoptotic index in the germinal layer [22]. These findings have led to the assumption that the host immune response could be the main factor driving CE cyst fertility [10,23,24].

Comparing wallabies and sheep CE cysts in Australia, Barnes, et al. [25] found differences between immune cells present in the adventitial layer of both animal hosts and in the intensity of the immune response. Therefore, cyst immune responses between different hosts could be of various immune responses, with variations in the immune cells in the adventitial layer of CE cysts. Regarding cattle and sheep CE cysts, there are clear adventitial-layer response patterns between them. Fertile cattle and sheep cysts are characterized by a thick fibrous capsule enveloping the laminated layer, with absence infiltrating immune cells, whereas non-fertile CE cysts show striking differences: in cattle, the laminated layer is surrounded by a granulomatous reaction, with abundant lymphoid follicles. Conversely, non-fertile sheep CE cysts show a fibrous capsule surrounding the laminated layer (like a fertile CE cyst), but it is infiltrated with a granulomatous reaction [24]. Therefore, the adventitial layer cannot be considered only a fibrotic reaction to the parasite tissue, but rather the expression of an active local host immune response.

Indeed, the adventitial layer is composed of different types of immune cells, such as macrophages, T and B cells, eosinophils, and fibroblasts [10,23,26]. Macrophages are found in cattle non-fertile CE cysts as both palisading and giant multinucleated cells, which are in contact with the laminated layer [10], whereas the macrophage population in sheep non-fertile CE cysts is more diffuse, as it is infiltrating the fibrous capsule instead of reacting to the laminated layer [24]. In addition to the immune cells present in the adventitial layer of CE cysts, molecules associated with these populations, such as calprotectin, may also play a role in the host response to parasitic infection. Calprotectin is a dimeric cytoplasmatic protein composed of S100A8 and S100A9, forming the S100A8/S100A9 complex. It is an antimicrobial protein involved in inflammatory regulation, cell proliferation, and cell differentiation, being expressed by monocytes, macrophages, and neutrophils, among other cell types [27]. As macrophages are part of granulomatous reaction and the adventitial layer of CE cysts, the calprotectin expression in situ in cattle and sheep remains unknown, as it has been determined only during in vitro experiments.

In mice experimentally infected with *E. granulosus* sensu lato, calprotectin expression determined by Western blot (WB), immunohistochemistry (IHC), and mass spectrometry (MS) was primarily associated with the cyst wall and was significantly more pronounced compared to CE cysts from other species, such as cattle and humans. In those species, calprotectin was less abundant than S100A12, another S100 protein [28]. Calprotectin has been studied in granulomatous reactions unrelated to CE cysts, such as infections caused by *Mycobacterium avium subspecies paratuberculosis*. In this context, calprotectin has been described as a cytosolic protein expressed in macrophages and has been used to identify this cell population in cattle tissue samples, along with other markers [29]. However, as its expression has been determined in mice experimental infection, which does not always represent the immune response from natural infection on *E. granulosus* hosts, the expression of calprotectin in cystic echinococcosis cysts from naturally infected hosts remains unknown.

Thus, the objective of this study is to completely characterize the calprotectin expression in the adventitial layer of cattle and sheep CE cysts in order to further understand the local immune response dynamics at play in both herbivore hosts. The analysis of CE naturally infected hosts with immunohistochemistry in tissue samples of CE cysts allows us to reach a more accurate approach to the immune response from natural hosts to the CE cyst development.

## 2. Results

### 2.1. Calprotectin Expression in CE Cysts from Cattle and Sheep

**Figure 1:**
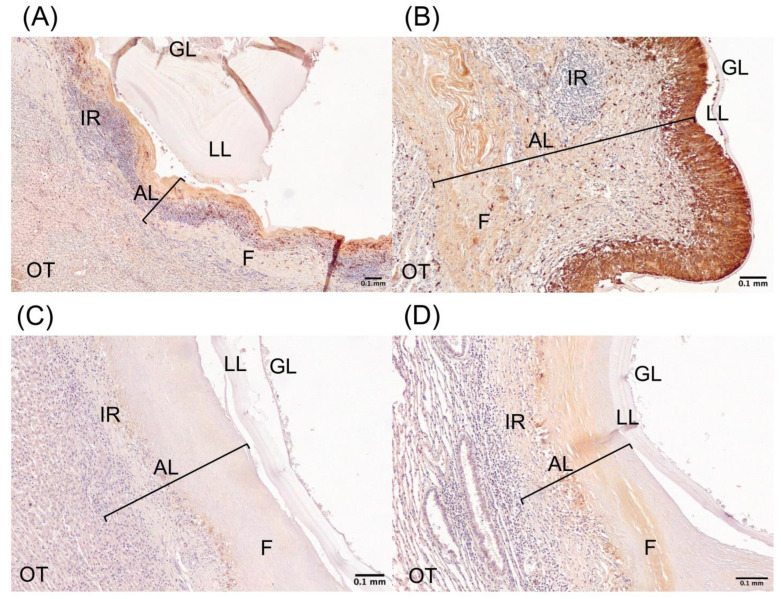
Calprotectin expression in cattle and sheep CE cysts. Representative images of immunohistochemistry (IHC) of calprotectin protein. Calprotectin IHC of (**A**) cattle fertile CE cyst, (**B**) cattle non-fertile CE cyst, (**C**) sheep fertile CE cyst, and (**D**) sheep non-fertile CE cyst. GL, germinal layer; LL, laminated layer; AL, adventitial layer (bracket line); IR, immune reaction; F, fibrosis; OT, organ tissue. Scale bar: 0.1 mm.

Cystic echinococcosis (CE) cysts from cattle and sheep were collected at abattoirs in different regions of Chile and transported to the laboratory for analysis. To compare calprotectin expression in the adventitial layer of CE cysts from cattle and sheep, immunohistochemistry with anti-calprotectin antibody was performed to determine calprotectin expression in fertile and non-fertile CE cysts (Figure 1 and Appendix A.2). In cattle, IHC of the adventitial layer shows the calprotectin expression forming a palisade in contact with the laminated layer of CE cysts (Figure 1A,B). However, this morphology is not seen in the calprotectin expression of the adventitial layer of sheep CE cysts, mostly forming a continuous line across the adventitial layer and in close contact with fibrosis (Figure 1C,D). This calprotectin IHC is the first approach specifically showing a difference between this antimicrobial expression on CE cysts from cattle and sheep.

**Figure 2:**
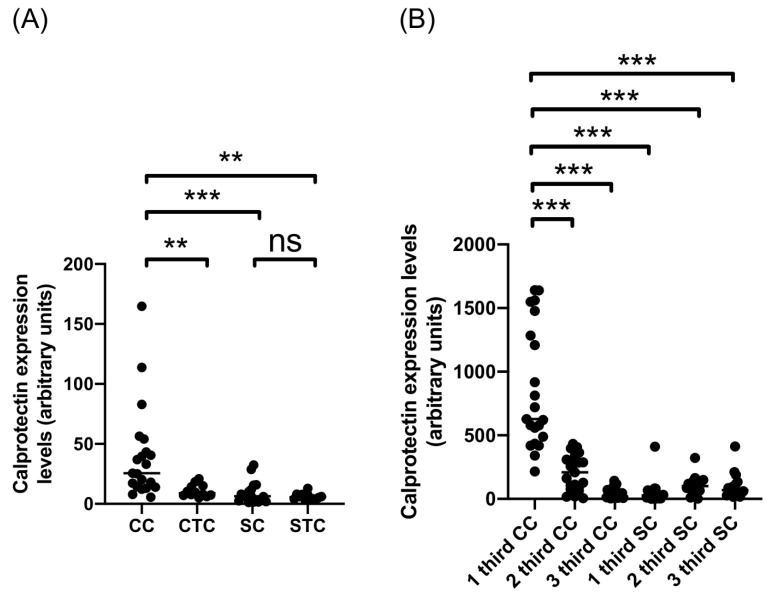
Expression levels of calprotectin in adventitial layer of CE cysts and tissue controls of cattle and sheep. Quantification of expression levels of calprotectin in (**A**) total adventitial layer and (**B**) thirds of adventitial layer of CE cysts and tissue controls from cattle and sheep. ** *p*-value < 0.01; *** *p*-value *<* 0.001; and ns, not significant. CC, cattle cyst; CTC, cattle tissue control; SC, sheep cyst; STC, sheep tissue control.

**Figure A1:**
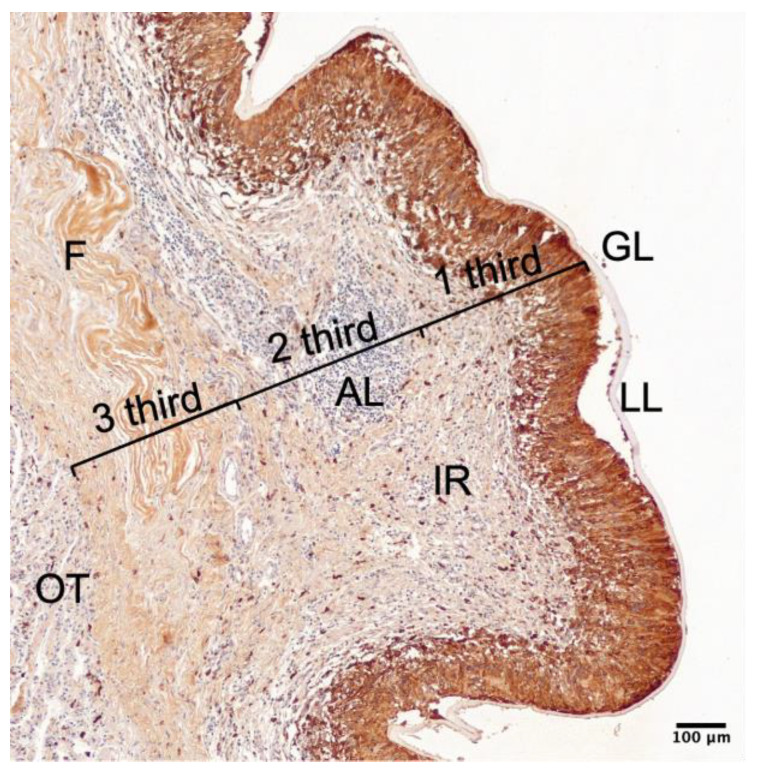
Segmentation of adventitial layer for immunohistochemical analysis. Adventitial layer of CE cysts was segmented into three sections (i.e., thirds). GL, germinal layer; LL, laminated layer; AL, adventitial layer; IR, immune reaction; F, fibrosis; OT, organ tissue. Scale bar: 0.1 mm.

Quantitative IHC analysis was used to further analyze the calprotectin expression in the adventitial layer of CE cysts from cattle and sheep (Figure 2 and Table 1). This analysis revealed significant differences between calprotectin expression in the adventitial layer of cattle and sheep CE cysts when all CE cysts were considered, regardless of fertility (Figure 2A). Also, the calprotectin expression was significantly increased in the adventitial layer of CE cysts from cattle compared to the cattle control tissue. In contrast, there was no difference between the adventitial layer of CE cysts from sheep and sheep tissue control (Figure 2A). This layer was divided into three sections to further analyze the differences in calprotectin expression within the adventitial layer of CE cysts, as shown in Figure A1. This segmentation allowed us to identify specific regions where calprotectin-expressing cells—such as palisading macrophages in cattle CE cysts—are predominantly located, as previously described by [10]. This was useful in the analysis to prevent the calprotectin expression dilution effect by the adventitial layer width. The same quantitative analysis was performed to determine calprotectin expression (Figure 2B). The calprotectin expression of the first section from the adventitial layer of CE cysts from cattle was significantly increased compared to the other two sections from cattle CE cysts and the three sections from the adventitial layer of sheep CE cysts (Figure 2B). These results show a significant difference between the calprotectin expression of cattle and sheep CE cysts.

### 2.2. Calprotectin Expression in Fertile and Non-Fertile CE Cysts from Cattle and Sheep

**Figure 3:**
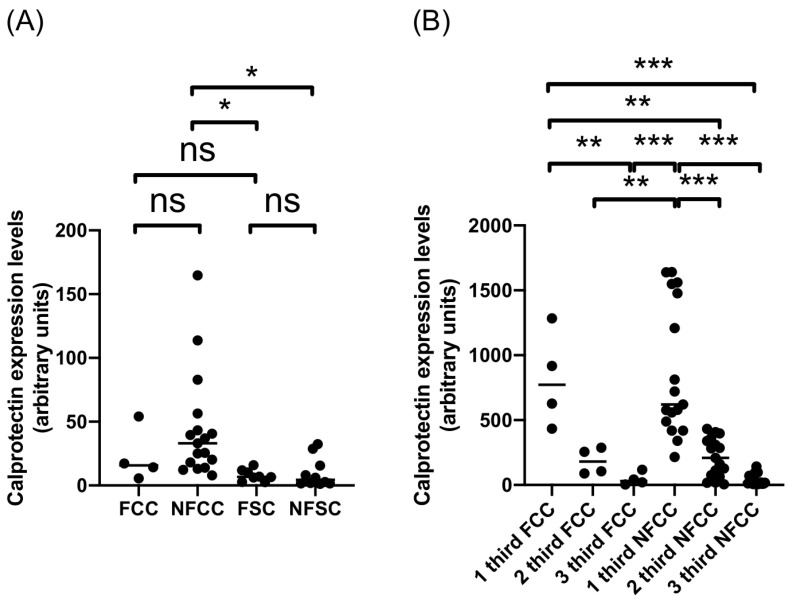
Expression levels of calprotectin in adventitial layer of fertile and non-fertile CE cysts from cattle and sheep. Quantification of expression levels of calprotectin in (**A**) total adventitial layer and (**B**) thirds of the adventitial layer of fertile and non-fertile CE cysts from cattle and sheep. * *p*-value < 0.05; ** *p*-value < 0.01; *** *p*-value *<* 0.001; ns, not significant. FCC, fertile cattle cyst; NFCC, non-fertile cattle cyst; FSC, fertile sheep cyst; NFSC, non-fertile sheep cyst.

To assess if this difference between calprotectin expression in the adventitial layer of CE cysts from cattle and sheep was related to CE cyst fertility phenotype, cysts were divided into fertile and non-fertile cysts (Figure 3 and Table 2). Calprotectin expression in the adventitial layer of non-fertile cattle CE cysts is significantly higher than that of fertile and non-fertile CE cysts from sheep (Figure 3A). Further analysis of adventitial layer sections in cattle CE cysts revealed a substantially higher calprotectin expression in the first section of non-fertile CE cysts compared to the other two sections (Figure 3B). However, in the case of fertile cattle CE cysts, the first section had a significantly higher expression of this protein than the third section, but not different from the second section, showing a more diffuse expression of calprotectin in these two sections (Figure 3B). The cross-analysis has shown that the first sections of either fertile or non-fertile cattle CE cysts are significantly higher than the two other sections of non-fertile and fertile CE cysts, respectively (Figure 3B). In the case of sheep CE cysts, the analysis revealed no significant differences between adventitial layer sections of fertile and non-fertile CE cysts, as seen in the comparison of the whole adventitial layer of both types of sheep CE cysts (Figure 3A). This analysis suggests that the fertility of CE cysts is not related to the difference in calprotectin expression in the adventitial layer of cattle and sheep CE cysts.

### 2.3. Calprotectin Expression in CE Cysts from Liver and Lung of Cattle and Sheep

**Figure 4:**
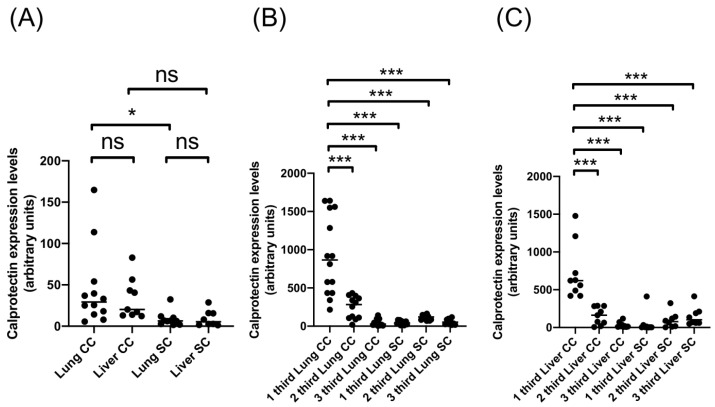
Expression levels of calprotectin in adventitial layer of CE cysts from liver and lung of cattle and sheep. Quantification of calprotectin expression levels in (**A**) total adventitial layer of CE cysts from liver and lung of cattle and sheep, (**B**) thirds of adventitial layer of lungs CE cysts and (**C**) thirds of adventitial layer of liver CE cysts. * *p*-value < 0.05; *** *p*-value < 0.001; ns, not significant; CC, cattle cyst; SC, sheep cyst.

Another cyst characteristic assessed in this study is the CE cyst’s location in cattle and sheep, which, in this case, was either lung or liver (Figure 4 and Table 3). Calprotectin expression was significantly higher in lung cattle CE cysts’ whole adventitial layer versus lung sheep CE cysts (Figure 4A). However, there was no difference between the adventitial layer of liver CE cysts from cattle and sheep (Figure 4A). Further analysis of adventitial layer sections showed the same pattern of significantly higher calprotectin expression in the first section of cattle CE cysts compared to all the other sections of cattle and sheep CE cysts from lung and liver origin (Figure 4B,C). These results suggest that CE cysts’ organ origin is unrelated to the difference between the calprotectin expression of the adventitial layer of CE cysts from cattle and sheep.

Our results reveal that calprotectin expression in the adventitial layer of CE cysts is significantly higher in cattle compared to sheep. This elevated expression is localized primarily in the inner section of the adventitial layer adjacent to the laminated layer, where palisading macrophages are present in cattle. In contrast, sheep cysts show a more diffuse and lower calprotectin signal. Importantly, these differences are not influenced by CE cyst fertility or organ location (liver vs. lung), suggesting that the host species could be the primary factor associated with the variation in calprotectin expression.

## 3. Discussion

CE cysts of *E. granulosus* s.s. from sheep are described to elicit a local immune response that forms a collagenous capsule in the adventitial layer that surrounds the parasite tissue. However, the same species of the parasite can infect other animal hosts, like cattle, as described in this work, where it generates a granulomatous reaction characterized by the presence of various immune cells [26], suggesting that CE cysts can differ in the composition of its adventitial layer between CE cysts from a different animal origin. The comparison of the adventitial layer of CE cysts from cattle and sheep showed variances in calprotectin expression, where cattle CE cysts had an increased expression of this protein compared to the sheep CE cysts. One study analyzed the walls of cattle-derived CE cysts using in vitro techniques such as mass spectrometry and Western blotting. In these experiments, the researchers evaluated the expression of S100 proteins, including calprotectin (the S100A8/S100A9 complex) and S100A12. S100A12 was found to be the most abundant protein in the bovine CE cyst wall, whereas calprotectin was scarcely expressed. In contrast, our results compared the expression of calprotectin (S100A8/S100A9) in fertile and non-fertile CE cysts from both cattle and sheep, revealing markedly higher calprotectin expression in cattle CE cysts compared to those from sheep [28].

As described previously, the macrophage population in cattle CE cysts exhibits a palisade morphology all along the laminated layer, and, in our results, this palisade shows high calprotectin expression within the context of the entire adventitial layer [10]. Conversely, in the case of sheep CE cysts, calprotectin expression is not only a fraction of that observed in cattle-derived cysts but also appears as a thin line adjacent to the fibrous reaction surrounding the laminated layer, as previously described [24]. Palisading macrophages are a characteristic hallmark of immunologic granulomatous reactions, and in cattle CE cysts, they are present as other immune cells that characterize granulomatous reactions like lymphocytes [10,23].

Differences between CE cysts from cattle and sheep have been analyzed recently. Moreover, other CE cysts characteristics have been analyzed, where they found significant differences between the thickness of parasitic layers (germinal and laminated layers) and the adventitial layer of CE cysts from cattle and sheep. Interestingly, they found giant cells in some CE cysts, a macrophage morphology related to the formation of granulomatous reactions [30]. Regarding PSC gene expression, there is a marked difference when those come from different animal origins. A study found 1364 genes differentially expressed between liver CE cysts from cattle and sheep [31]. Concomitantly, the germinal-layer cells and PSCs produce exosomes and extracellular vesicles that carry proteins such as antigen B2 and TSPAN14 [32], and non-coding RNAs involved in mediating the inflammatory response and the collagen catabolic process [33], which could modulate these differential CE cyst phenotypes.

In the classification of CE cysts from cattle and sheep in terms of the order of the components of its adventitial layer, cattle CE cysts are described as an immunologic granulomatous reaction; however, sheep CE cysts are described as a fibrous reaction that could or not have an inflammatory response distal to the cyst, if they are non-fertile or fertile, respectively [24]. In this case, the analysis of calprotectin expression shows that in fertile and non-fertile CE cysts, the magnitude of this antimicrobial expression has no significant differences, suggesting that calprotectin is always present in fertile and non-fertile CE cysts. This is also the case of cattle CE cysts, where the fertility of the cyst is not related to the magnitude of calprotectin expression.

Regarding CE cyst location, we found no significant differences in the magnitude of calprotectin expression between liver or lung cysts in cattle nor sheep. This effect was also suggested in the case of the organization of the adventitial layer, where the order of the inflammatory reaction and the fibrous reaction had no significant differences when compared to CE cysts from different organs [24].

In cattle, the higher calprotectin expression associated with granulomatous responses correlates with predominantly non-fertile cysts, positioning cattle as epidemiological “dead-end” hosts with limited contribution to parasite transmission. In contrast, sheep, despite showing lower calprotectin expression and a primarily fibrotic adventitial response, harbor mostly fertile cysts, which sustain the life cycle through the release of viable protoscoleces to infect canids [24]. These host-specific patterns also imply that treatment strategies may need adjustment: in sheep, fibrosis and fertility may necessitate prolonged benzimidazole therapy, while in cattle, reduced fertility could justify less aggressive dosing. Recently, systemic calprotectin has been proposed as a biomarker in infectious diseases [34], so future research should evaluate macrophage polarization and drug pharmacodynamics across host species, which may ultimately guide tailored dosing strategies that optimize efficacy while minimizing unnecessary treatment.

Macrophage polarization helps to respond to different stimuli and function as an inflammatory cell in its classical activation or M1 activation or to function as a reparatory or regulatory cell in its alternative activation or M2 activation [35]. This polarization may be related to the role of macrophages in the local granulomatous response, in which there is a transition from a Th1 response to a Th2 response [36]. Evidence found that *Echinococcus granulosus* s.l. infection might cause the polarization of macrophages to M1 or M2 [37,38,39,40,41] activation. Thus, evaluating the polarization of the macrophage populations in the adventitial layer of cattle and sheep CE cysts is necessary.

## 4. Materials and Methods

### 4.1. Samples and Processing

**Figure 5:**
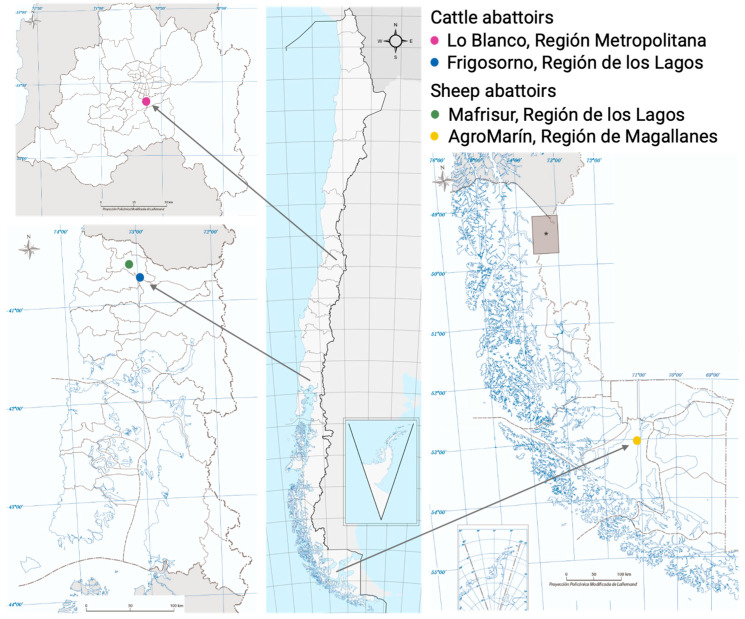
Geographical location of cattle and sheep abattoirs included in this study. *: Balmaceda’s airport for geographical reference.

Cystic echinococcosis cysts from cattle and sheep were obtained in abattoirs from different locations in Chile. Since sheep production is mostly located in the south of Chile, our samples were obtained from abattoirs located in both Osorno, Región de los Lagos and Punta Arenas, Región de Magallanes. In contrast, cattle production has a more disperse distribution, so samples were obtained both from the metropolitan area, as well and from the southern regions (Figure 5).

As previously described [24], viscera with CE cysts went through official veterinary inspection. We received their help to obtain each CE cyst without opening it, preferably cutting the parenchyma from liver or lung, and each CE cyst was packed individually. The selection of animals that were going to be sampled was dependent firstly on the presence of other parasites or infection in abattoirs. If there were no other parasites than *E. granulosus* s.l., samples were collected and transported to the laboratory. Next, each CE cyst was processed individually with a syringe to extract hydatid liquid, a scalpel to obtain wall CE cyst fragment, and a cotton swab for DNA sample. In this process, if samples showed caseification, calcification, or hemorrhagic content, they were not included in the study.

CE cysts were separated by cyst location and fertility, the latter assessed by trypan blue exclusion test observed under a light microscope. For this study, 39 CE cysts were included: 21 cattle CE cysts and 18 sheep CE cysts. Cattle CE cysts were subdivided as follows: 4 fertile CE cysts (1 from liver and 3 from lungs) and 17 non-fertile CE cysts (8 from liver and 9 from lungs). Sheep CE cysts were subdivided as follows: 8 fertile CE cysts (3 from liver and 5 from lungs) and 10 non-fertile CE cysts (5 from liver and 5 from lungs).

### 4.2. Immunohistochemical Procedure

CE cysts’ sections were embedded in paraffin using an automatic tissue processor, and paraffin blocks were then cut in a microtome to obtain 3 mm thick sections for IHC protocol. To evaluate calprotectin expression in the adventitial layer of CE cysts, calprotectin immunohistochemistry was performed as previously described, but with [10] modifications. Briefly, tissue sections were deparaffinized, and then antigen retrieval with trypsin 0.05% was performed at 37 °C for 15 min. The primary antibody for calprotectin (Invitrogen S100A9 Monoclonal Antibody MAC387, Carlsbad, CA, USA) was incubated overnight at 4 °C at a dilution of 1:100 for cattle CE cyst sections and 1:50 for sheep CE cyst sections. Then, tissue sections were incubated with ImmPRESS^®^ HRP universal antibody (MP-7500, Vector Laboratories, Burlingame, CA, USA) for 2 h at 37 °C. Detection was performed with a DAB-Plus Substrate Kit (Life Technologies, Waltham, MA, USA), and then tissue sections were counterstained with hematoxylin. Cattle and sheep control tissue was performed by quantification of IHC on liver and lung tissue where there was no CE cyst.

### 4.3. Imaging and Analysis

Tissue sections were imaged in a FSX100 Inverted Microscope (Olympus, Abington, MA, USA) and a Leica DM3000 with Microvisioneer Camera and Software (Leica Microsystems, Wetzlar, Germany). For the analysis of images, 0.5 µm^2^ tissue fragments were obtained, and semi-quantification was performed as described by [42]. Briefly, deconvolution of image fragments was performed using Fiji ImageJ 2.14, and then quantification was performed following instructions. Statistical analysis was performed by ANOVA analysis with Bonferroni post hoc test in GraphPad Prism 8.

## 5. Conclusions

This study demonstrated that calprotectin expression in the adventitial layer of *Echinococcus granulosus* sensu stricto cysts is significantly higher in cattle compared to sheep. This difference was localized to the inner adventitial zone adjacent to the laminated layer and was independent of cyst fertility or organ location. These results indicate that the host species is a key determinant of local immune responses to CE cysts. A brief consideration is that such host-specific differences may influence parasite fertility and, consequently, epidemiological patterns, but further studies are required to confirm functional implications.

## Figures and Tables

**Table 1:** Expression levels of calprotectin in adventitial layer of CE cysts and tissue controls of cattle and sheep.

Study Group	Calprotectin Expression Levels ^1^	Number of Samples
Cattle CE cysts	39.92	21
1 third CC	861.7	21
2 third CC	207.2	21
3 third CC	39.61	21
Cattle tissue control	10.86	12
Sheep CE cysts	6.378	16
1 third SC	56.57	16
2 third SC	110.3	16
3 third SC	96.98	16
Sheep tissue control	6.140	12

^1^ Calprotectin expression levels are expressed in arbitrary units, and the number for each line corresponds to the mean of samples for each group.

**Table 2:** Expression levels of calprotectin in adventitial layer of fertile and non-fertile CE cysts from cattle and sheep.

Study Group	Calprotectin Expression Levels ^1^	Number of Samples
Fertile cattle CE cysts	22.78	4
1 third FCC	815.9	4
2 third FCC	184.1	4
3 third FCC	46.10	4
Non-fertile cattle CE cysts	43.96	17
1 third NFCC	872.5	17
2 third NFCC	212.6	17
3 third NFCC	38.08	17
Fertile sheep CE cysts	7.890	8
1 third FSC	33.05	8
2 third FSC	102.5	8
3 third FSC	120.0	8
Fertile sheep CE cysts	4.865	8
1 third NFSC	80.08	8
2 third NFSC	118.1	8
3 third NFSC	73.92	8

^1^ Calprotectin expression levels are expressed in arbitrary units, and the number for each line corresponds to the mean of samples for each group.

**Table 3:** Expression levels of calprotectin in adventitial layer of CE cysts from liver and lung of cattle and sheep.

Study Group	Calprotectin Expression Levels ^1^	Number of Samples
Lung cattle CE cysts	55.36	12
1 third lung CC	921.8	12
2 third lung CC	246.6	12
3 third lung CC	44.85	12
Liver cattle CE cysts	12.21	9
1 third liver CC	727.0	9
2 third liver CC	154.6	9
3 third liver CC	32.63	9
Lung sheep CE cysts	1.589	9
1 third lung SC	43.45	9
2 third lung SC	117.7	9
3 third lung SC	63.89	9
Liver sheep CE cysts	1.226	7
1 third liver SC	67.08	7
2 third liver SC	100.7	7
3 third liver SC	139.5	7

^1^ Calprotectin expression levels are expressed in arbitrary units, and the number for each line corresponds to the mean of samples for each group.

**Figure A2:**
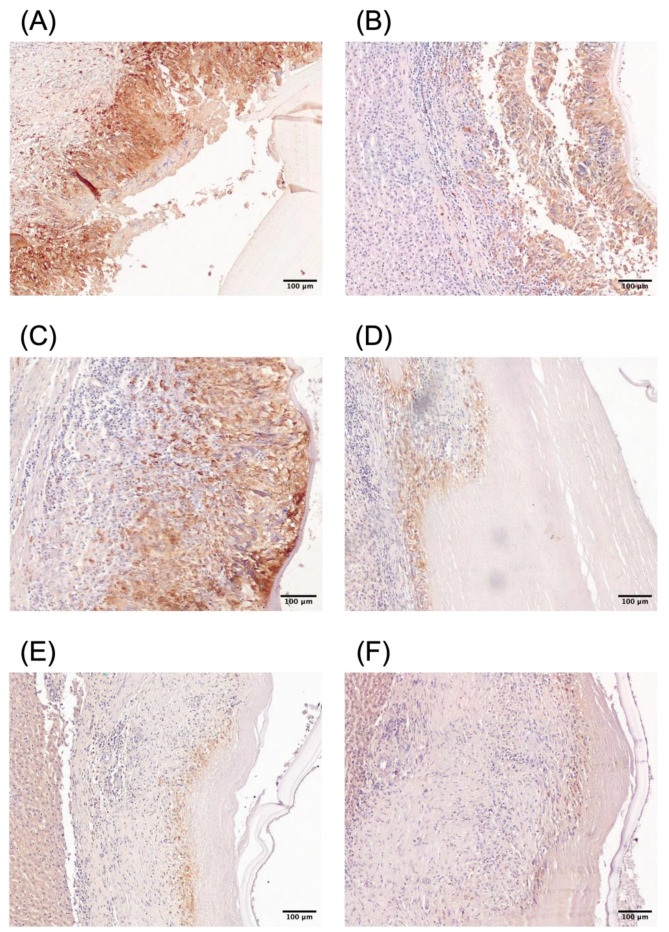
Samples of adventitial layer from CE cysts of cattle and sheep. Microphotographs from adventitial layers of cattle and sheep CE cysts from other samples that were used in this study. (**A**) Fertile lung, (**B**) non-fertile liver, and (**C**) non-fertile lung adventitial layer from cattle CE cysts. (**D**) Fertile liver, (**E**) non-fertile liver, and (**F**) non-fertile liver adventitial layer from sheep CE cysts. Scale bar: 100 μm.

## Data Availability

The original contribution presented in this study is included in the article. Further inquiries can be directed toward the corresponding author.

## Abbreviations

The following abbreviations are used in this manuscript:

CE	cystic echinococcosis
s.s.	sensu stricto
s.l.	sensu lato
PSCs	protoscoleces
WB	Western blot
IHC	immunohistochemistry
MS	mass spectrometry
AL	adventitial layer
LL	laminated layer
GL	germinal layer
IR	immune reaction
F	fibrosis
OT	organ tissue
CC	cattle cyst
CTC	cattle tissue control
SC	sheep cyst
STC	sheep tissue control
ns	non-significant
FCC	fertile cattle cyst
NFCC	non-fertile cattle cyst
FSC	fertile sheep cyst
NFSC	non-fertile sheep cyst
